# SARS-CoV-2, recurrent ischemic strokes and carotid macrothrombosis: the other face of the cytokine storm (a case report)

**DOI:** 10.11604/pamj.2021.38.34.27645

**Published:** 2021-01-13

**Authors:** Jehanne Aasfara, Said Jidane, Aziza Laarje, Khalid El Yamani, Lahcen Belyamani, Youssef Tijani

**Affiliations:** 1 Department of Neurology, Cheikh Khalifa International University Hospital, Faculty of Medicine, Mohammed VI University of Health Sciences (UM6SS), Casablanca, Morocco,; 2 Emergency Department, Mohamed V Military Hospital, Mohamed V University of Rabat, Rabat, Morocco,; 3 Department of Cardiology, Cheikh Khalifa International University Hospital, Faculty of Medicine, Mohammed VI University of Health Sciences (UM6SS), Casablanca, Morocco,; 4 Department of Intensive Care, Cheikh Khalifa International University Hospital, Faculty of Medicine, Mohammed VI University of Health Sciences (UM6SS), Casablanca, Morocco,; 5 Department of Vascular Surgery, Cheikh Khalifa International University Hospital, Faculty of Medicine, Mohammed VI University of Health sciences (UM6SS), Casablanca, Morocco

**Keywords:** SARS-CoV-2, ischemic stroke, cytokine storm, macrothrombosis, case report

## Abstract

Since December 2019, the world has experienced the emergence in China of a new infection called severe acute respiratory syndrome coronavirus 2 (SARS-CoV-2). This infection quickly has progressed to a global pandemic since March 2020, with very high human-to-human transmission rate. Besides lung injury, COVID-19 is also associated with cardio and neurovascular complications. Herein, we report the case of a 77-year-old female who presented with non-severe COVID-19 and multiple ischemic strokes secondary to an extensive carotid thrombosis. The ischemic stroke was supposed to have been caused by the cytokine storm related to COVID-19. The possibility of hemorrhagic transformation, based on the assessment of bleeding score, limited the use of anticoagulation, and probably explained the stroke recurrence and poor outcome in our patient. The pathogenic mechanism and the management of this complex situation are still lacking and further studies are needed.

## Introduction

Since December 2019, the world has experienced the emergence in China of a new infection responsible of fatal pneumonitis due to a virus belonging to the *Coronaviridae* family, called severe acute respiratory syndrome coronavirus 2 (SARS-CoV-2). This infection quickly has progressed to a global pandemic since March 2020, with very high human-to-human transmission rate [1]. Besides lung injury, COVID-19 is also associated with cardio and neurovascular complications. We here report the case of a non-severe COVID-19 patient showing multiple ischemic strokes secondary to an extensive carotid thrombosis associated to the disease.

## Patient and observation

**Figure 1:**
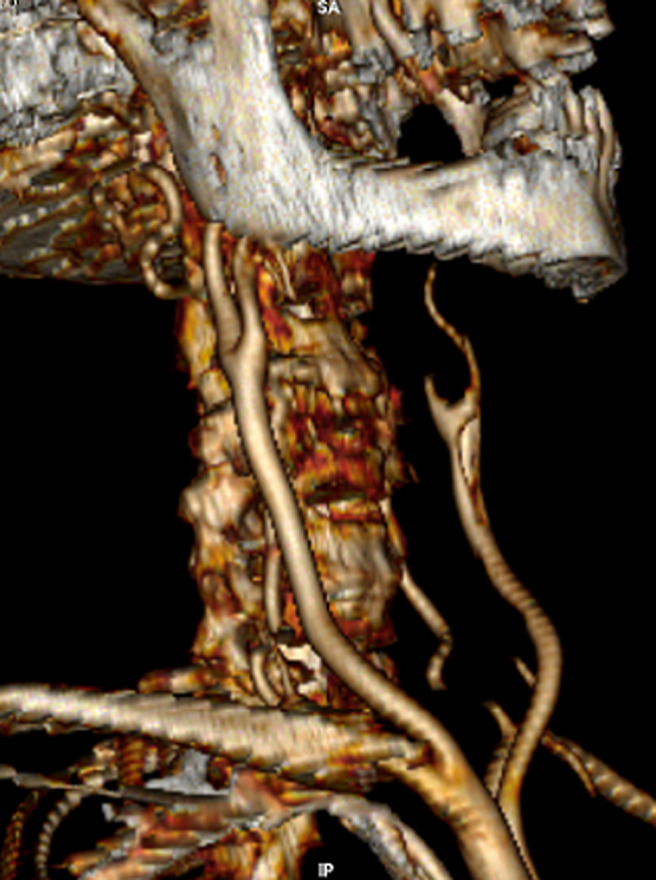
computed tomography angiography of cervical vessels shows thrombosis within the right common carotid artery extending to the internal carotid artery

A 77-year-old female with a medical history of treated hypertension, developed 24 hours before admission a sudden onset of right facial droop, right hemiplegia and aphasia with a national institute of health stroke scale (NIHSS) score of 16. Magnetic resonance imaging (MRI) demonstrated a large acute infarct in the left middle cerebral artery (MCA) territory. Doppler ultrasound showed an occlusion of the left internal carotid artery and the computer tomography (CT) angiography of supra-aortic trunks revealed thrombosis within the left common carotid artery extending to the internal carotid artery with no evidence of underlying arterial dissection (Figure 1). A family member reported that she had also experienced a dry cough and diffuse myalgia a few days ago, without fever or dyspnea. Given the pandemic context, a reverse-transcriptase polymerase chain reaction test for SARS-CoV-2 was performed before her admission to a non-COVID sector of our hospital and was positive. The chest CT was performed and was unremarkable. Blood tests results revealed an elevated C-reactive protein at 26.20mg/L, a high ferritin at 2061mg/mL, D-dimer at 1000.03mg/mL and lymphopenia at 0.9 x 10^3^cells per mL. Platelets count and LDH were normal. Evaluation of underlying causes of stroke was performed, and all tests, including screening for antiphospholipid antibodies, were unremarkable.

**Figure 2:**
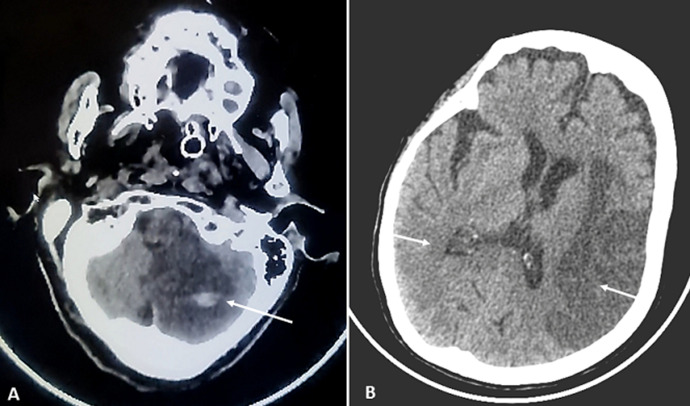
axial head computed tomography shows: A) infarction of the left cerebellar hemisphere with hemorrhagic transformation; B) acute ischemic infarction in the distribution of the right middle cerebral artery and sub-acute left middle cerebral artery territory infarction

Electrocardiogram (EKG) was normal, and transthoracic echocardiography demonstrated left ventricular hypertrophy without intracardiac thrombus or right-to-left shunting. The patient was treated with aspirin and prophylactic anticoagulation with enoxaparin (4000UI/24h) because she was outside the licensed time window for thrombolysis and thrombectomy. She also received Azithromycin, Vitamin C, Vitamin D and Zinc for SARS-Cov-2 infection. Four days after her admission, she developed mild renal insufficiency and presented a generalized tonic-clonic seizure treated with levetiracetam. Neurological examination revealed left facial paralysis and left hemiplegia. Control CT scan showed a new large hypo attenuated area within right MCA and hemorrhagic transformation of left cerebellar infarct (Figure 2). Because of the high risk of bleeding, anticoagulation treatment was stopped. The patient´s condition gradually deteriorated during her hospitalization and developed respiratory distress complicated by consciousness disorders leading to her admission to the intensive care unit (ICU). The patient died a week later due to respiratory and circulatory failure.

## Discussion

Herein, we report a macro-vascular thrombosis presenting as large vessel stroke in a patient with non-severe COVID-19. Li Y *et al*. reported that 5% of patients with severe SARS-CoV-2 infection developed stroke with a median age of 71.6 years and had a higher incidence of cardiovascular risk factors [2]. Stroke occurs after an average time of 10 to 12 days after SARS-CoV-2 diagnosis whereas our patient presented with stroke in early stages of her illness with a mild clinical presentation and without major risk factors [3]. This suggests that even non-severe cases of COVID-19 can be associated with stroke and macrothrombosis, a fact that was previously reported by Fara *et al*. in three cases of stroke secondary to large-artery thrombosis [4]. It is well known now that the SARS-CoV-2 can generate an inflammatory cytokine storm with hypercoagulability, platelet activation, microcirculatory endothelial damage [5] and macroangiopathy probably due the virus-endotheliun interaction [6]. Indeed, SARS-CoV-2 targets primarily lung cells by binding to the transmembrane angiotensin- converting enzyme 2 (ACE2) protein. Besides type 2 pneumocytes, SARS-CoV-2 can enter other host cells expressing angiotensin converting enzymes 2 such as endothelial cells, cardiomyocytes, pericytes, and macrophages. These cells subsequently lose antigiotensin converting enzymes 2 activity leading to reduced angiotensin II inactivation and decreased conversion to angiotensin which triggers platelet aggregation, vasoconstriction and increased thrombogenicity leading to recurrent thromboembolic complications [7].

The management of stroke due to carotid macrothrombosis in COVID-19 patients is based on anticoagulants. However, the choice of anticoagulant, the dose and potential drug interactions with COVID-19 pharmacotherapy make the therapeutic decision very challenging, especially in the presence of significantly high risk of bleeding. In the absence of clear recommendations on this topic, it has been suggested to use preferably unfractionated heparin than low-molecular-weight heparin as anticoagulant. Early initiation of therapeutic anticoagulation can reduce thromboembolism in patients with ischemic stroke associated to SARS-CoV-2 infection [8]. We suppose that in our case, the cytokine storm related to COVID-19 caused large-vessel ischemic stroke. Additionally, our patient had a high risk of hemorrhagic transformation (advanced age, large infarct volume, renal impairment) which limited the use of anticoagulation and explained probably the stroke recurrence and poor outcome.

## Conclusion

Our case highlights the possibility of association between SARS-CoV-2, macrothrombosis in the internal carotid artery and stroke as a presenting symptom of the disease even in non-severe COVID-19. Preliminary data indicate that angiopathic thrombosis can be induced by viral endotheliopathy or endotheliitis but other pathogenic mechanisms and the management of this complex situation are still lacking and further studies are needed.
